# On the Distinction between Analytical and Synthetical Judgments

**Published:** 1861-07

**Authors:** C. M. Ingleby


### Art. II.—ON THE DISTINCTION BETWEEN ANALYTICAL AND SYNTHETICAL JUDGMENTS.

By C. M. Ingleby, LL.D.

If it be asked from whom, *par excellence*, do modern metaphysicians derive the substance and method of their systems? the answer must be Immanuel Kant. Slips of his Critical Philosophy have been from time to time grafted upon our native stocks, philosophical and theological, and have borne fruit. Yet even to reflecting minds, Kant's masterpiece—that work which was the result of twelve years' earnest intellectual toil, and was not commenced till he had attained the maturity of his unrivalled intellect—is a sort of *Ultima Thule* or *Timbuctoo* in philosophy. From a distance men speculate as to the philosophical whereabouts of the *Kritik der reinen Vernunft*, and wonder what it is: and some, having learned the reports of the adventurous few who have set foot on the charmed region, assume a familiarity with its matter and method, and glibly smatter the outlandish shibboleth of Kant. By these pretenders the *Critic of Pure Reason* is sometimes spoken of as a system of Subjective Idealism; while among the outsiders who make no pretence, the Sage of Königsberg is regarded as a dreamer who wrote a book to prove that man can scale the heavens by means of a faculty of pure reason. Whereas the fact is, that Kant's system is essentially realistic, and its end may be summed up in one sentence—Speculative Reason is legitimately employed only upon the objects of sense.

The *Critic of Pure Reason* is doubtless a difficult book—1st, on account of the novelty of its method; 2ndly, on account of its thoroughness in enumerating the elements, and expounding the processes of Reason; and 3rdly, on account of the technicalities in which its doctrines are expressed, or rather *involved*. Of these, (1) is an ample justification of (3): and as to (2), it may be sufficient to remark, that if, as Dr. Johnson said, "all shallows are clear," it is equally true that profundity, especially in mental science, is necessarily an occasion of obscurity to the uninitiated, just as the depth of a ravine seems to the distant beholder lost in haze and shadow. All deeps are obscure—till they are penetrated.

On the threshold of the *Critic of Pure Reason* we meet with one crucial doctrine, the misapprehension of which is a bar to further progress. Yet here how many expositors of Kant have been unable to find the clue! It was here that Cousin made a capital error in his *Leçons sur Kant*. The foundation-stones of all science (physical or metaphysical) are judgments. The understanding is the faculty of judging (according to the senses), and its functions are (1) Conception; (2) Judgment; and (3) Reasoning. The act of judgment is the affirmation (or negation) of *some relation* between two concepts—a concept being the result of that operation of distinct thought which is called Conception. Ordinary logic is conversant with one kind of relation only—viz. Identity. It deals with concepts only as regards their respective quantities, and their relation to one another, as contained and containing. The proposition in ordinary logic simply affirms or denies that *so much* of one concept is identical with *as much* of another concept. With a concept as *such*, logic has no concern. Thus: when it is said that *All men are mortal*, Logic regards only the three incidents of the proposition, (1) the expressed quantity "all" (universal) of "men," and the implied quantity "some," (indefinite) of "mortal," (2) the quality of the proposition, viz., that it is affirmative, and (3) the relation of the quantified concepts ("all men," "some mortals") to each other—viz., that "all men" are identically the same persons as "some mortals;" or, what is the same thing, that "mortal" is predicate of "all men." With the concepts "men," "mortal," logic has no concern—that is, with their matter or meaning. Accordingly we may express an affirmative proposition in the three general forms, all A is B, some A is B, A is B, according as A is taken universally, indefinitely, or individually. It must be distinctly comprehended that in each of these propositional forms, A (as also B) may stand for a determinate quantum of knowledge obtained through any of the channels through which it is to be acquired. Now, though logic has no concern with the matter of these concepts, yet in metaphysics (as in physics) account, and very special and exact account, is taken of their content. If Logic demands *How much of them is thought?* Metaphysics inquires *What do they stand for?* Now for all purposes of judgment A and B stand for *concepts* and for concepts only. A, for instance, may stand for a sensation, a perception, an intuition, a reminiscence, an idea, or a feeling—but from what source soever it may be derived, *into a judgment it can enter only as a concept.* Putting quantity, for the present, out of the question, we may say generally that, when it is affirmed that A is B, A and B are concepts only.

Judgments, as to their matter, are of two kinds, judgments *à priori*, and judgments *à posteriori*. There is a school of metaphysics in which the very possibility as well as the actuality of judgments *à priori* is denied. I shall not pause to refute that school; I will merely say that the *à priori* character of several classes of judgments can be substantiated on evidence as demonstrative as that of any proposition in Euclid. Judgments *à priori*, though coming with, and, in fact, partly constituting our reflective experience, do not depend upon experience; *e.g.*, *Two straight lines cannot enclose a space. The three angles of a triangle are together equal to two right angles.* The criteria of such judgments is their *necessity* and *universality*. We can readily perceive the *impossibility* of two straight lines enclosing a space, and as readily the *impossibility* of the three angles of a triangle being greater or less than two right angles; *i. e.*, we perceive the necessity of the two judgments just instanced. We can likewise see that those propositions are universally true—that, in point of fact, they are not true of such straight lines and triangles as observation, through the senses, can afford us; for such lines have breadth, and are not absolutely straight, and such triangles are bounded by three such approximately straight broad lines; but that they are, nevertheless, true of all *conceivable* straight lines and triangles which the reason can find, through imagination, in the intuition of space.

On the other hand, judgments *à posteriori* not only come with experience, but actually owe their truth to experience, and to experience only, and are only true so far as experience has verified them. Such as, *The sun will rise to-morrow. Roses are either red or white. All animals that divide the hoof are ruminants.* These propositions, if true, are true solely on the strength of our observation on nature.

In the *Introduction* to the *Critic of Pure Reason* (§ iv.) Kant (having premised the distinction I have just drawn) expounds another distinction in judgments, which is as fundamental as the last, and, moreover, so essential to the very existence of the *Critic*, that it may be safely asserted that the reader who is vanquished by any difficulty here will obtain no passport into the region beyond. It is the *pons asinorum* of the Critical Philosophy. Let us see whether it cannot be made plain to all readers of reflection. Kant says—

"In all judgments wherein the relation of a subject to a predicate is thought . . . . . . . . this relation is possible in two ways. Either the predicate B belongs to the subject A, as something which is contained (though covertly) in the concept A, or the predicate B lies completely out of the concept A, although it stands in connexion with it. In the first case I call the judgment *analytical*, in the second *synthetical*. Analytical judgments (affirmative) are, therefore, those in which the connexion of the predicate with the subject is thought *through identity* [in conception]; those in which this connexion is thought *without identity* [in conception] are called synthetical judgments. We might call the former *explicative*, the latter *ampliative* judgments; since the former add, by means of a predicate, nothing to the concept of the subject, but only separate it by analysis into its constituent concepts, which were already thought in the subject, although [perhaps] in a confused manner; the latter add to our concept of the subject a predicate which was not at all thought in it, and which [therefore] by no analysis [of that concept] could have been discovered therein."

Kant has been "soundly rated" for his style; and I confess it is possible to produce from the *Critic of Pure Reason* samples of syntactical construction which are barbarous (see *Trans. Æsth.* § 9, ii. for specimens ready to hand).* Yet I doubt if it were possible to mend the foregoing statement. It is precise, explicit, and brief. Is it intelligible? A very short acquaintance with the *Critic* served to give me a probable clue to the sense of this passage; and eleven years' familiarity with Kant's works has served to convince me that the clue I then obtained was all-efficient. It is, however, a fact within my own knowledge, that readers of the *Critic* generally carry away an inaccurate and one-sided impression from Kant's exposition of the distinction between analytical and synthetical judgments.

The first danger is, that the reader will understand nothing about it, but that is comparatively unimportant. The second and greater danger is, that he (or she, for I know one female student of Kant) will superficially understand the foregoing extract, and in metaphysics "a little knowledge" is worse than absolute ignorance. He might carry away either of two impressions.

1. That a judgment is synthetical only where the predicate is not actually expressed in the subject. From this definition it would follow that *A triangle is a figure that has only three angles* is an explicative judgment (which indeed it is), but that *A plane triangle is a figure bounded by three straight lines* is an ampliative judgment (which it is not).

2. That a judgment is analytical wherever the predicate can be discerned *by any process* to belong to the subject. According to this definition, it would follow that *Two straight lines cannot enclose a space* is an explicative judgment; for, if it be expressed thus, *Enclosed space is always bounded by more than two straight lines,* it is not difficult to perceive, by an act of reason, that the predicate must always belong to the subject. It would also follow that an ampliative judgment is in fact nothing else than a judgment which we *cannot* discern to be true, for the moment

* Here is one, which I give in Mr. Heywood's travestied version—

"Everything which is represented by a sense, is so far at all time phenomenon; and an internal sense ought therefore not at all to be admitted, or the subject which is the object of this could be represented by the same (*sense*) only as phenomenon, and not as it (the subject) would judge of itself, if its intuition were simply self-effectivity, that is, intellectual." Kant, however, must not be made to bear the blame of this.

we discover its truth, the predicate belongs to the subject. (Whereas the fact is, that the axiom just enunciated is an ampliative (or synthetical) judgment.)

In the first place, let us consider what an explicative (or analytical) judgment is. It is necessary and universal, and therefore *à priori.* It is founded solely on "the principle of contradiction" (or "the principle of non-contradiction," as the late Sir W. Hamilton called it). If, in the judgment, A is B, the predicate B is contained in the subject A (*i.e.*, as a part of the concept A), it is plain that the judgment is quite independent of any appeal to the testimony of the senses. For, to deny the proposition A is B (which is to deny the predicate B of A) when in fact we already know (and understand) that A contains the predicate B, is to identify the opposed propositions, A is not B, and A is B, which is a violation of one of the laws of thought.*

Ampliative judgments, like explicative judgments, are formuled by the understanding in harmony with its own laws, and therefore of that law which is called "the principle of contradiction;" *but the truth of an ampliative judgment does not depend upon that principle*—for, in denying the truth of such a judgment, we are not involved in a direct contradiction in thought. If the judgment be *à priori,* its denial is simply repugnant to the intuition (Space or Time) from which we derived it. If the judgment be *à posteriori,* its denial is but a denial of the testimony of the senses, or of an inference founded on that testimony.

All judgments, *à posteriori,* then, are ampliative.

Now let us sum up the relations of judgments as distinguished, on the one hand, by the *matter,* and on the other by the *form* of their concepts, *i.e.,* as *à priori—à posteriori,* and as *explicative —ampliative.*

| | |
|---|---|
| The class "ampliative judgments consists of | All "judgments *à posteriori,*" and Some "judgments *à priori.*" |
| All "explicative judgments" and Some "ampliative judgments" | Constitute the class "judgments *à priori.*" |

Assuming that by this time my reader understands *formally* what an explicative judgment and what an ampliative judgment means, I will proceed to inquire under what *material* conditions a proposition, A is B, expresses an explicative judgment. Kant says it does so if A and B are concepts, and "the predicate B belongs to the subject A, as something which is contained (though covertly) in the concept A." Now it might be supposed that in order to determine whether the predicate B is contained—however implicitly—in the concept A, it is necessary to predetermine the

* On this point, see the K. R. V., Anal. II. § 1.

character of the *term* or *name* A, whether it is *denotative* or *connotative*—*i.e.*, whether it is merely a mark of recognition (as a numbered label on a picture in the Royal Academy Exhibition), or whether it is a name expressing the properties of the concept (as the description of that picture in the catalogue). But in fact this inquiry is not even logical—it is merely grammatical. In metaphysics we have nothing to do with the art of naming—there we are concerned wholly and only with the matter of the concepts.

Now how are we to determine the content of the concept A? Of course, if that name be thoroughly connotative, our work is done for us; but if not we must go to the concept, and analyse it as we are conscious of its content. How then do we express the result of that analysis? By *definition*. A concept may be defined in any of three ways: (1) by its extension; (2) by its intension; or, (3) by any of its distinctive properties; of which (2) only gives us the content of the concept. Hence it appears that we may express the result of an analysis of the concept A, in the form of an intensive definition. (*Intension*, by the way, is a generic term employed to indicate the properties of any object of thought as they form the part or whole of a given concept). We are not called upon to enumerate all the properties of the object, but only those represented by the concept. When we say A is B (*i.e.* affirm B of A), A is an *ens rationis*, and may be more or less exhaustive of the properties of the object conceived. If A be an *inadequate* concept, it might be hardly possible to form any true judgment on A that was not an ampliation of it. If A be a *complex* concept, it might be hardly possible to form any judgment on A that was not an explication of it. And this Kant himself implicitly admits to be the case when he speaks of the concept of *rectitude* (*Trans. Æsth.* § 91). "No doubt the concept of *right*, as employed by a sound understanding, contains all that the most subtle speculation can evolve from it, though in the ordinary and practical use we are not conscious of the manifold representations (given) in that thought." But it does not follow that the Idea of Rectitude cannot be the subject of an ampliative judgment, or else the terms Ampliative (Synthetical) and Explicative (Analytical) must be used in a relative sense; whereas, Kant employs them for a material and *qua* absolute distinction.

Let us consider how we conceive objects *à priori* and *à posteriori*. When an object is presented to the senses we form a rough concept of it, which we will call A. Yet, vague and inadequate as this concept is, it comprises all our knowledge about the object. Suppose now that, by examination of the object, a new fact concerning it comes to our knowledge. Let this addition to our knowledge be expressed thus, A is B, and the resulting concept of the object be written A B. Here the judgment A is B is clearly ampliative *à posteriori*. If we obtain further knowledge of the object, in the shape of predicates, C, D, E, . . . &c., we successively form the judgments, A B is C, A B C is D, A B C D is E, . . &c. All of these are ampliative *à posteriori*. That is all that Kant means when he says that all judgments of experience are synthetical, the reason of which he thus states, "it would be absurd to found an analytical judgment on experience, because, in framing such a judgment, I have no occasion to go out of my concept, nor, by consequence, to have recourse to the testimony of experience."

We have seen how it comes to pass that our judgments of experience modify our concepts of external objects, so that it is impossible to fix the concept as a standard of reference in any judgment. Such an inconvenience, however, does not obtain in judgments *à priori*. Concepts *à priori* are originally simple. Space and Time are apprehended in their integrity, and not like objects of the senses, piecemeal. Space is accordingly called by Kant a pure *intuition*, (anschauung) so also is Time. The simple concept of either of these intuitions (or *projections*, as I should prefer calling them) is (or should be) the same for every man. If in my mind the concept be complex, I am called upon to denude my concept of everything adventitious:—*i.e.* everything that has accrued to my original concept by acts of the judgment of faculty, until I arrive at *a concept which cannot be defined by analysis of its content*. The concept has a content; indeed it contains the simple integer (Space or Time) which has been revealed to the consciousness immediately on intuition, and *that which is revealed in its integrity is necessarily undefinable.* Hence the futility of the attempt of Coleridge and others to define "Life." This is obvious, "Life" is a concept *à priori*, and by consciousness is revealed in its integrity and not by its accidents. But intensive definition can only proceed by analysis of the concept to be defined. Thus it follows that "Life" is not intensively definable. So of all concepts *à priori*. And for the very reason that they cannot be defined, the only explicative judgment that can be formed of them is the co-ordinate judgment, A is A. All such judgments are, of course, analytical *à priori*, and consequently all other judgments wherein the undefinable concept A is the subject must be ampliative. Thus, *Space is of three dimensions*, is an ampliative judgment. The simple concept of Space is the reflex of the single perception of Space, which is the ground of sensuous experience. The predicate here is not contained in the concept. Accordingly we must go out of the concept *into the intuition*, and learn by the use of reason upon the representation, that Space has the property of triplicity of dimension. For this reason, our judgment thereupon is a synthesis, and is rightly called synthetical, or ampliative.

Analytical or explicative judgments, though not extending the sphere of knowledge, are yet, as Kant says, very important and necessary as guaranteeing clearness to our concepts. In fact, they lead to intensive definitions of concepts, which, by their very character, are purely explanatory of the subjects of those judgments.

In this place I may, by way of illustration, call attention to the fundamental error of M. Cousin, to which I have already adverted. He writes (I quote from Mr. Henderson's translation) —"It is necessary to distinguish between the axioms of geometry and its true principles. The first are purely analytical . . . The axioms . . . are indispensable, but unproductive . . . the true geometrical principles are the definitions [those of a triangle, a circle, and a straight line, are instanced] which are synthetical *à priori* judgments." This is not only a slip-shod statement, but contains a fatal blunder. Euclid's so called definitions of a triangle, a circle, and a straight line, are not homogeneous. If Euclid's definition of a triangle be taken as his type of a definition, his definition of a straight line is an axiom, and that of a circle is virtually a theorem. *A plane triangle is a figure bounded by three straight lines,* and *A rectilineal is a line that is straight,* are homogeneous propositions, and are rightly called definitions, on the one hand, of a *triangle,* on the other of a *rectilineal,* and both express explicative judgments. *A plane figure that is bounded by three straight lines has the fewest possible number of sides,* and *A line that is straight is placed symmetrically with respect to the space on both sides of it* are also homogeneous propositions, and are rightly called axioms, being both expressive of ampliative judgments and susceptible of being made the basis of a deductive system.

From these considerations it is evident that M. Cousin has not properly discriminated between axioms and definitions in geometry. But he has been guilty of worse than confusion; he has actually reversed their relative values; for geometry owes no more to its definitions than it does to a dictionary, and everything to its postulates and axioms. In geometry, every judgment must be derived from a representation, or construction in space. But those that are explicative merely are easily discriminated from those which are ampliative; for the former owe their *identity* to a construction merely, whereas the latter owe their *synthesis* to an inference which Reason draws from a construction in Space. Thus, *A triangle is a figure of three sides,* is explicative of the representation of a triangle, as it subsists in the subject; but

*A triangle has the sum of its angles equal to two right angles,* is a judgment of the synthesis of the subject "triangle," and affirms a predicate which is not found in that concept; and that synthesis is an inference from the representation of "triangle." *

In algebra (including arithmetic), according to Kant, all our judgments are ampliative *à priori.* Thus 5 + 4 = 9, and v + v′ = V, are alike ampliative judgments. The question is, do we find in the concept 9 the synthesis of 5 and 4? No; for all we find in the concept 9 are *nine units,* and these we cannot distribute in any form (as 8 + 1, 7 + 2, 6 + 3, or 5 + 4), without having recourse to intuition of number (as given in Time), or to observation on a manifold object (as our hand); in truth, the very fact that in the higher numbers we have to resort to concrete objects (as ciphers), in default of the power of mental arithmetic, proves that we do not possess the synthesis of two numbers in the concept of one: else why need we calculate at all? And these remarks apply, *mutatis mutandis* to quantity, as distinct from number.

While assenting to the validity of Kant's distinction between explicative and ampliative judgments, it may be permitted to any student of his philosophy to doubt as to the propriety of any particular illustration or example. Now in the *Prolegomena to Every Future Metaphysic* (a sort of defence of the previous work, the *Critic of Pure Reason*), Kant gives, *Gold is a yellow metal,* as an example of an explicative (or analytical) judgment. Against which proceeding I take leave to cite Kant's own remarks in his former and greater work, viz., "it would be absurd to found an analytical judgment on experience;" yet sure enough, if *Gold is a yellow metal* be an analytical judgment, it is one that is founded on experience; for it is only by experience that we learn the colour (not constant) of that precious metal which we denominate *gold.* In fact, while *Gold is a metal* and *Body is extended* are homogeneous, both being explicative; *Gold is yellow* and *Body is heavy,* are also homogeneous, both being ampliative; indeed, *Body is extended* and *Body is heavy* are cited by Kant in the *Critic* as examples, the one of an explicative and the other of an ampliative judgment.

Kant seems to have been under the impression that he first expounded and discriminated analytical and synthetical judgments. He does, in fact, in the *Critic of Pure Reason,* point out how near Hume was to grasping the distinction between them; and in the *Prolegomena* he cites "the third head of the

---

* I do not charge Cousin with confounding explicative with ampliative judgments. He sees clearly enough that the latter must be at the foundation of science. He says, "Les vrais principes géométriques sont des lois synthétiques *à priori.*" That is right enough; but his application of it is wrong.

fourth book" of Locke's *Essay on the Human Understanding* as containing a hint of this division of judgments. But, as he says, Locke's remarks are so vague and so little reduced to rules that the distinction hinted at was not likely to attract notice. Now had Kant been acquainted with the works of the "good" Buffier, (as that writer would probably, from his ecclesiastical character, have been designated by the Königsberg Professor), he would have found that he had been anticipated in his celebrated division of judgments by the Frenchman, and that in the most express manner; for Buffier not only clearly discriminates between these two kinds of judgments, but denominates the one kind *Identical* and the other *Conjunctive,* a nomenclature which has some advantages over that generally employed by Kant. The preceding discussion being quite independent of Kant's special expositions, may possibly contain error; but the writer conceives that even if such should turn out to be the case, the doctrine of Kant has in this manner a better chance of being intelligible to English readers, than if it had (according to unvarying precedent) been presented in the prescriptive phraseology of the great teacher himself.

